# iValiD-TB: a fully characterized *Mycobacterium tuberculosis* dataset for antimicrobial resistance bioinformatics workflow validations

**DOI:** 10.3389/ftubr.2024.1441923

**Published:** 2024-09-13

**Authors:** Pascal Lapierre, Joseph Shea, Shannon G. Murphy, Carol Smith, Donna Kohlerschmidt, Michelle Dickinson, Kimberlee A. Musser, Vincent Escuyer

**Affiliations:** ^1^Wadsworth Center, New York State Department of Health, Albany, NY, United States; ^2^The Johns Hopkins University School of Medicine, Baltimore, MD, United States

**Keywords:** tuberculosis, *Mycobacterium tuberculosis*, whole genome sequence (WGS), bioinformatics, clinical validation, antimicrobial susceptibility, antibiotic resistance

## Introduction

Tuberculosis has been a bane of humanity for centuries and, still to this day, is estimated to affect more than two billion people worldwide (1). The slow growth rate of *Mycobacterium tuberculosis* (MTB) is a major challenge for timely diagnosis and appropriate treatment of cases (2, 3). Diagnostic delays can lead to increased disease burden, cost increases and risk of treatment failures (4). A crucial aspect for clinical assay validations is the availability of well-characterized samples to assess the specificity and sensitivity of the methodology being tested (10). Due to logistical constraints, the nature and availability of specimens, and geographic diversity of the strains, many laboratories struggle with access to adequate clinical MTB specimens for their validation needs (11). Consequently, validation studies may not include representative of the myriad of clinical samples and drug resistant profiles that a clinical laboratory may receive. Therefore, diverse and well-characterized datasets for standardized next generation sequencing (NGS) assay validations for MTB NGS tests are needed. Reference datasets of clinical TB samples and synthetic genomes were released in the past with limited phenotypic drug susceptibility testing (DST) information for research, development and proficiency testing purposes (5, 6). Here, we have assembled a comprehensive dataset of well-characterized whole genome sequences (WGS) from *Mycobacterium tuberculosis* strains to aid in the development of clinical assays for this pathogen. This dataset includes complete whole genome sequences paired-end read sets obtained through Illumina MiSeq sequencing, along with detailed profiles of drug susceptibility patterns and mutations known to be associated with antimicrobial resistance (AR) to nine MTB drugs. This dataset has been curated to be inclusive of a broad range of lineage diversity, drug susceptibility profiles, and mutation types. As such, this dataset only contains two separate pairs of strains that are phylogenetically closely related (iValiD-TB-S22 and iValiD-TB-S23 with 0 SNP differences and iValiD-TB-S6 and iValiD-TB-S46 with 6 SNPs differences) based on our pipeline results. A complete SNP matrix has been included in Supplementary Table 3. The sequence reads dataset has been made available for bioinformatics pipeline development, and for clinical assay validation of the bioinformatic analysis pipeline, serving as a valuable resource to advance research and enhance the development of clinical MTB NGS assays.

**Figure 1:**
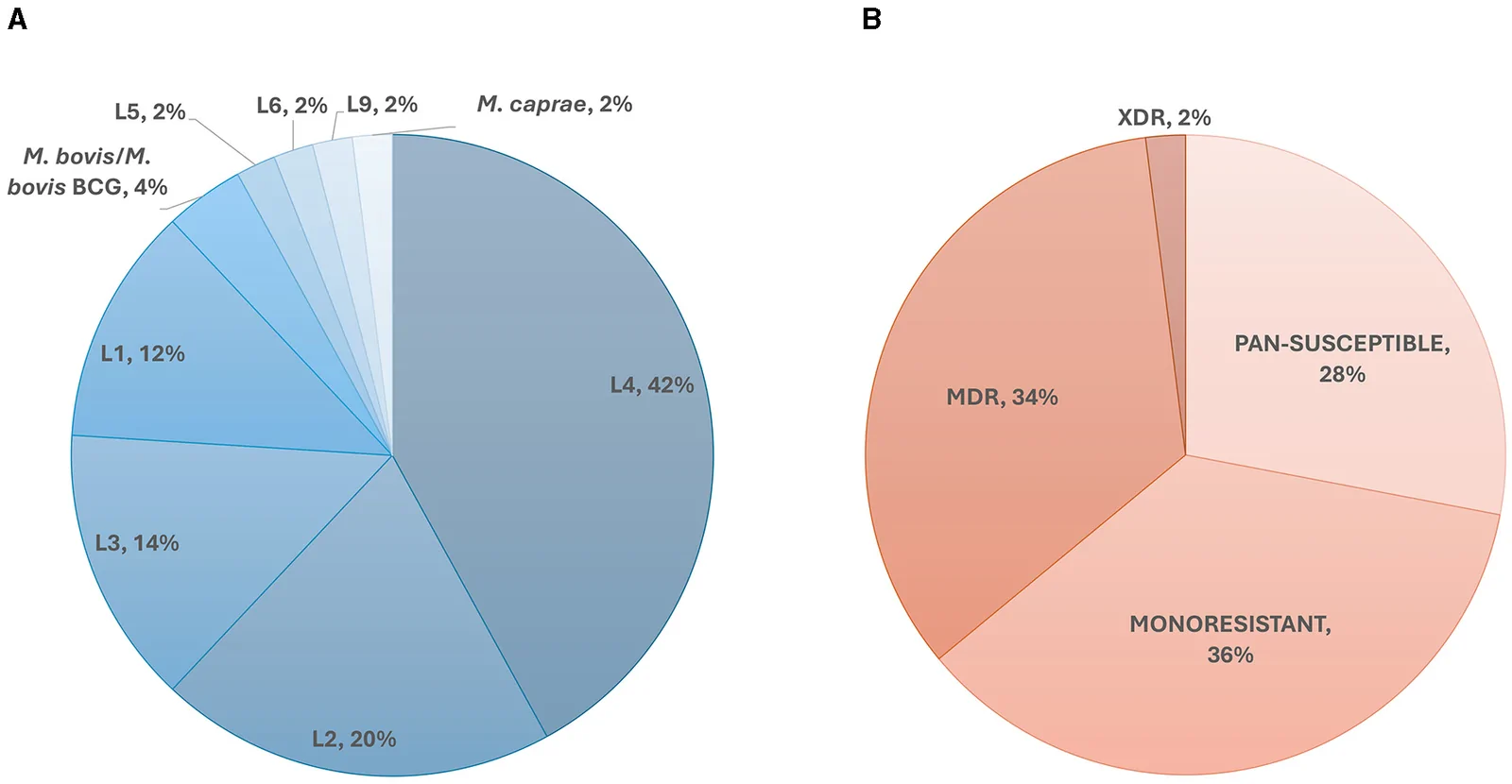
Overall dataset composition. **(A)** Lineage and species diversity of the dataset. The species identifications were confirmed by real-time PCR and through Kraken as described in Shea et al. (7). The lineages were determined *in-silico* using lineage-specific genomic markers. **(B)** Phenotypic drug susceptibility representation of the samples in our dataset. MDR, Multidrug-Resistant; XDR, Extensively Drug-Resistant.

**Figure 2:**
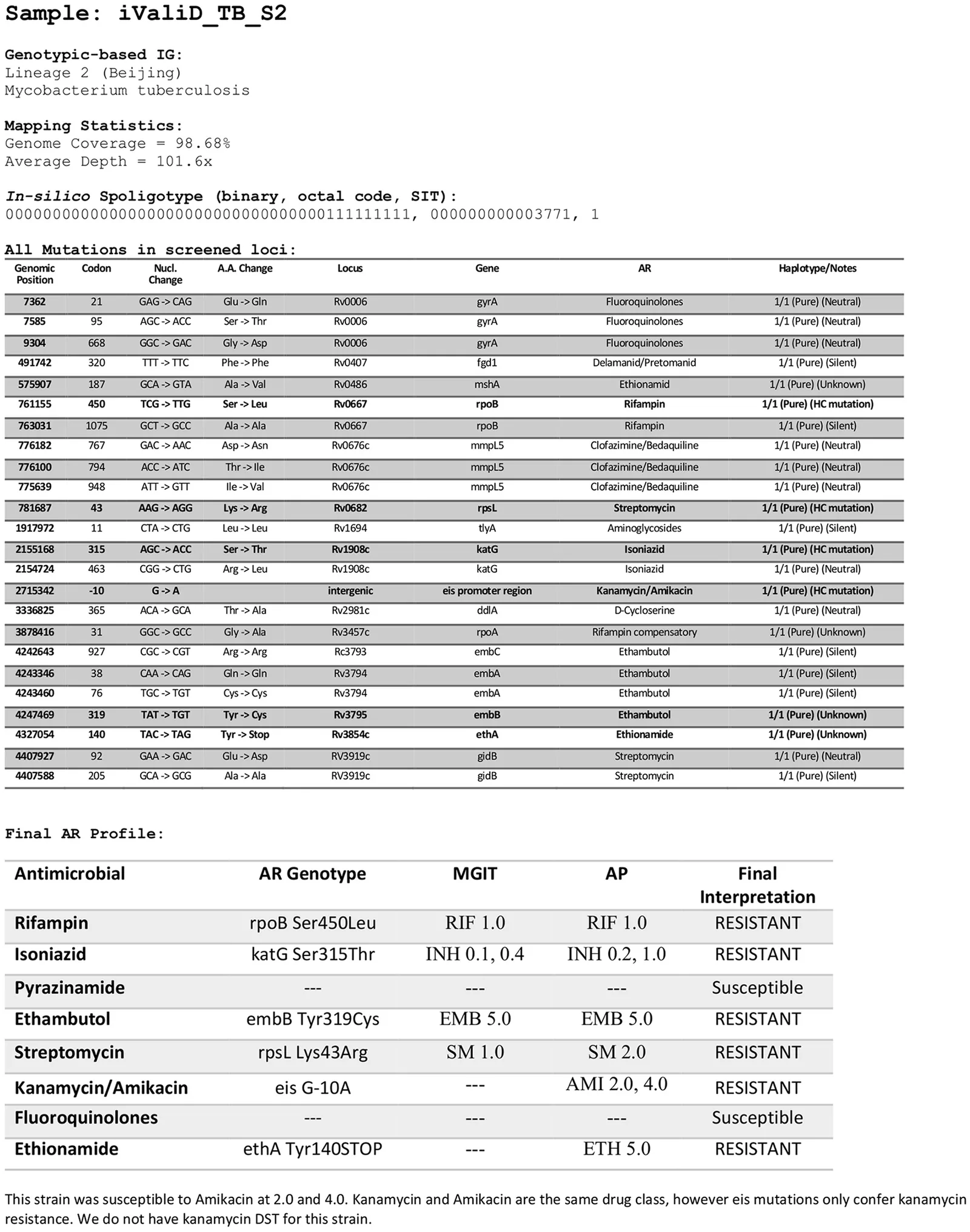
Sample report card. Example of report card for one of the samples in the dataset listing the locations of the mutations, mutation types, drug susceptibility testing results, the final interpretation of the drug resistance, lineage information, spoligotype, and expected mapping metrics. The report cards are available in Dryad at: https://doi.org/10.5061/dryad.4j0zpc8m8. RIF, Rifampin; INH, Isoniazid; EMB, Ethambutol; SM, Streptomycin; AMI, Amikacin; ETH, Ethionamide, PZA, Pyrazinamide; FQ, Fluoroquinolones. DSTs concentrations are in μg/ml.

A total of 50 members of the *Mycobacterium tuberculosis* complex (MTBC) were sequenced, which includes 47 strains of *Mycobacterium tuberculosis*, one *Mycobacterium caprae* strain, one *Mycobacterium bovis* strain and one *Mycobacterium bovis*-BCG strain (Figure 1). These strains were part of our collection of samples obtained from New York State patients since the implementation in 2013 of our clinical diagnostic and reporting TB NGS assay (7). Of the MTBC, 6 strains are from Lineage 1, 10 from Lineage 2, seven from Lineage 3, 21 from Lineage 4, and 1 representative of each of Lineages 5, 6, and 9. Of the 50 samples, 14 were determined to be pan-susceptible to nine drugs (rifampin, isoniazid, ethambutol, pyrazinamide, streptomycin, ethionamide, fluoroquinolones, kanamycin, and amikacin) by phenotypic drug susceptibility testing, 18 were mono-resistant, 17 multi-drug resistant (MDR) and one was extensively drug resistant (XDR; Figure 1, Supplementary Table 1). A total of 1,073 different mutations present in the 53 screened loci (Supplementary Table 2) characterized by WGS in this dataset, of which, 107 mutations were identified to be associated with drug resistance, most of which are part of the World Health Organization 2023 Catalog of mutations in *Mycobacterium tuberculosis* (8). The New York State Department of Health implemented this assay for clinical diagnostic before the WHO catalog was released and as such, we are using our own susceptibility interpretation criteria. Consequently, the users of this dataset will have to use their own decision criteria based on their individual workflow characteristics and interpretations. The characterized mutations included single nucleotide polymorphisms (SNP), stop codons, promotor mutations, small insertion and deletions (indels), and large genomic deletions. The locations of the mutations, types, effect on drug resistance, as well as DST results, lineage information, spoligotype and expected mapping statistics are all listed in an individual report card for each sample (Figure 2). The release of this fully characterized dataset will facilitate the development and benchmarking of bioinformatics tools for MTB NGS diagnostics and aide in the validation of these clinical assays. The read sequences are accessible from the NCBI SRA Bioproject PRJNA980174. The associated AR reports cards for the 50 samples are available in Dryad at: https://doi.org/10.5061/dryad.4j0zpc8m8.

## Methods

Genomic DNA extraction, sequencing library preparation and bioinformatics pipeline methods were described in Shea et al. (7). DSTs were determined by either the agar proportion method on solid 7H10 agar or Becton Dickinson 960 system MGIT SIRE-P assay according to the Clinical and Laboratory Standards Institute's recommendations (9). The following concentrations were used for DST determinations: Streptomycin 1.0 μg/ml, Isoniazid 0.1, 0.2, 0.4, and 1.0 μg/ml, Rifampin 1.0 μg/ml, Ethambutol 5.0 and 10.0 μg/ml, Pyrazinamide 100 μg/ml, Kanamycin 5.0 μg/ml, and ofloxacin (1.0, 2.0, and 4.0 μg/ml). Ofloxacin is used in our laboratory as a representative of the fluoroquinolone (FQ) drug class. Genotypic identification, mapping statistics and *in-silico* spoligotyping were done as described in Shea et al. (7).

## Data Availability

The datasets presented in this study can be found in online repositories. The data is available in Dyrad at: https://doi.org/10.5061/dryad.4j0zpc8m8.
